# Therapeutic Effects of MOTS-c in the Valproic Acid–Induced Autism Model in Rats: Role of Tetrahydrobiopterin and Brain-Derived Neurotrophic Factor

**DOI:** 10.1007/s12035-026-05741-y

**Published:** 2026-02-18

**Authors:** Sıla Güvenir Seven, Hakan Sahin, Gözde Erkanlı Şentürk, Nesibe Uysal, Hafize Uzun, Oğuzhan Ekici, Gafur Rakıcı, Gönül Şimşek

**Affiliations:** 1 Department of Physiology, Cerrahpaşa Faculty of Medicine, Istanbul University-Cerrahpaşa, Istanbul, Turkey; 2 Department of Histology and Embryology, Cerrahpaşa Faculty of Medicine, Istanbul University-Cerrahpaşa, Istanbul, Turkey; 3 Cerrahpaşa Faculty of Medicine, Istanbul University-Cerrahpaşa, Istanbul, Turkey; 4 Department of Medical Biochemistry, Faculty of Medicine, Istanbul Atlas University, Istanbul, Turkey; 5 Department of Physiology, Faculty of Medicine, Çanakkale Onsekiz Mart University, Çanakkale, Turkey; 6 Department of Physiology, Faculty of Medicine, Istanbul Medeniyet University, Istanbul, Turkey; 7 Department of Physiology, Faculty of Medicine, Istanbul Atlas University, Istanbul, Turkey

**Keywords:** Autism spectrum disorder, MOTS-c peptide, Oxidative stress, Prefrontal cortex, Purkinje cells

## Abstract

Autism spectrum disorder (ASD) is a neurodevelopmental disorder characterized by impaired social interaction and repetitive behaviors, with currently limited therapeutic options. Oxidative stress is suggested as significant in ASD pathophysiology, making antioxidant strategies a promising therapeutic direction. Exercise reduces oxidative stress, alleviates ASD symptoms, and increases tetrahydrobiopterin (BH4) and brain-derived neurotrophic factor (BDNF) levels through AMP-activated protein kinase (AMPK) activation. MOTS-c, a mitochondrial-derived peptide acting through AMPK, mimics the effects of exercise but reportedly does not cross the blood-brain barrier (BBB). Considering the challenges in exercise adherence in ASD, our study hypothesizes that MOTS-c could increase circulating BH4 and BDNF, both of which are BBB-permeable, and alleviate oxidative stress and ASD symptoms. To evaluate this hypothesis, we investigated the effects of MOTS-c in the valproic acid–induced rat model of autism. Pregnant Sprague-Dawley rats received intraperitoneal 500 mg/kg valproic acid or saline on embryonic day 12. Female and male offspring were treated with 0.5 mg/kg/day MOTS-c or saline intraperitoneally from postnatal days 21 to 46. Following behavioral testing, animals were sacrificed, and histological and biochemical analyses were performed. Valproic acid exposure led to impaired sociability, repetitive behaviors, anxiety, cerebellar Purkinje cell loss, and increased oxidative stress and neuronal damage in the prefrontal cortex. These alterations were reversed by MOTS-c, except for anxiety and neocortical damage. No significant changes in plasma BH4 or BDNF levels were detected. Through its neuroprotective and antioxidant effects independent of BH4 and BDNF, MOTS-c may alleviate autism-like behaviors, suggesting its potential as a therapeutic candidate for ASD.

## Introduction

Autism spectrum disorder (ASD) is a neurodevelopmental disorder characterized by persistent deficits in social communication and interaction, along with repetitive patterns of behavior [1]. According to the last report published by the Centers for Disease Control and Prevention, ASD affects 1 in 31 children (3.22%) in the USA, with a 3.4-fold higher prevalence in males [2]. Taken together with previously published reports, it is evident that the prevalence of ASD has increased over the years [2–6]. Compared with males, females diagnosed with ASD may have better social communication skills and less pronounced repetitive behaviors. In addition to behavioral differences, various studies have shown some pathophysiological and molecular differences between the sexes [1, 7].

The pathophysiological and etiological mechanisms of ASD are not fully understood. The majority of cases are classified as idiopathic, with no specific cause identified. Studies have indicated that a variety of genetic (including single-gene mutations, copy number variations, and polygenic risk factors) and environmental (such as maternal immune activation and prenatal toxin exposure) factors may contribute to the pathogenesis of this disorder [8, 9]. Postmortem and neuroimaging studies have suggested that the behavioral abnormalities observed in ASD may be associated with neuroanatomical alterations, particularly in the prefrontal cortex (PFC), hippocampus, and cerebellum. Additionally, numerous studies have reported that neuroinflammation and oxidative stress may be involved in the pathophysiology of ASD [10].

Since the pathophysiology of ASD has not been fully elucidated, no curative treatment is currently available. Pharmacological treatments (such as antipsychotics, antidepressants, and anxiolytics) and nonpharmacological supportive therapies are applied to manage accompanying symptoms. Although these pharmacological treatments are often effective in addressing certain behavioral problems, such as aggression, self-injurious behavior, and anxiety, they do not eliminate the core symptoms of autism [8, 11–13].

Various animal models have been developed to elucidate the pathophysiology of ASD and explore potential treatment strategies. Some of these models are genotype-based, whereas others are environmentally induced. The valproic acid (VPA)–induced autism model was first established by Rodier et al. in 1996 [14]. Subsequent studies have investigated its construct, face, and predictive validity [12, 15, 16]. VPA (or 2-propylpentanoic acid) is a short-chain fatty acid that is commonly prescribed for epilepsy treatment or as a mood-stabilizing agent. VPA affects gene expression by inducing chromatin remodeling through histone deacetylase inhibition and modulates neurotransmission. However, VPA is teratogenic in humans, and clinical studies have demonstrated that prenatal VPA exposure is associated with neural tube defects, congenital malformations, developmental delays, cognitive impairments, and autism. The risk of ASD is approximately threefold greater in children exposed to VPA in utero. In rodents, prenatal VPA exposure results in neuroanatomical and behavioral alterations that closely resemble human autism. Therefore, the VPA model is widely acknowledged as a well-validated and frequently utilized rodent model for investigating the underlying neurobiology of idiopathic autism and exploring potential therapeutic approaches [8, 12, 17].

MOTS-c (mitochondrial open reading frame of the 12S rRNA-c) is a mitochondrial-derived peptide composed of 16 amino acids and is encoded by a small open reading frame located in the 12S rRNA-coding region of mitochondrial DNA [18]. Studies on its effects on cellular pathways have demonstrated that MOTS-c inhibits the folate/methionine cycle at the 5-methyltetrahydrofolate level, leading to an increase in the levels of 5-aminoimidazole-4-carboxamide ribonucleotide (AICAR), a potent activator of adenosine monophosphate-activated protein kinase (AMPK) [19]. AMPK, an enzyme present in all mammalian cells, is associated with multiple metabolic pathways. AMPK functions as an energy sensor of cells, becoming activated under low-energy conditions, thereby inducing cellular pathways that promote adenosine triphosphate production. Through this mechanism, AMPK is recognized as a key regulator of cellular energy homeostasis and metabolic balance in the body [20]. Additionally, MOTS-c has been reported to translocate to the nucleus under metabolic stress conditions, where it binds to chromatin and regulates the gene expression of NRF2 (nuclear factor erythroid 2-related factor 2), a key transcription factor involved in antioxidant defense. This effect of MOTS-c is AMPK dependent, and through this mechanism, MOTS-c has been shown to enhance cellular resistance to stress [21].

Exercise has been reported to increase AMPK expression in skeletal muscle in humans, as well as in skeletal muscle, adipose tissue, and the liver in rodents. AMPK is thought to be responsible for many of the beneficial effects induced by exercise [22]. MOTS-c is considered an “exercise mimetic” peptide due to its physiological effects, which are mediated by increased AICAR levels and AMPK activation [19, 23, 24].

Studies investigating the effects of exercise on ASD have demonstrated that specific exercise interventions significantly improve social communication and motor skills while reducing stereotypical behaviors. Among the nonpharmacological treatment approaches for autism, exercise is considered one of the most prominent [11, 13, 25, 26]. However, it has been reported that the physical activity levels and adherence to exercise programs of children with autism are considerably lower than those of their healthy peers, because of the difficulties they experience in social communication and motor skills [13]. Moreover, accumulating evidence suggests that oxidative stress is increased in autism [27–29]. These findings lead to the hypothesis that MOTS-c, a peptide with both “exercise-mimetic” and antioxidant properties, may have a therapeutic role in autism. Consequently, in our study, MOTS-c administration was planned in a rat model of autism. However, since MOTS-c has been reported to be unable to cross the blood-brain barrier (BBB) [30], our study proposes that MOTS-c may exert its effects through alternative molecules capable of crossing the BBB in the periphery.

AMPK leads to an increase in tetrahydrobiopterin (BH4) and brain-derived neurotrophic factor (BDNF) levels, both of which have positive effects on oxidative stress [31, 32]. Similarly, exercise has been reported to increase BH4 and BDNF levels via AMPK activation while reducing oxidative stress in the brain [33–35]. Moreover, studies have indicated that BH4 and BDNF levels are decreased in autism [36, 37].

On the basis of these findings, we hypothesized that the MOTS-c peptide, which mimics the effects of exercise, may increase the circulating levels of BH4 and BDNF through AMPK activation and that these molecules, both of which are known to cross the BBB [38, 39], could reduce oxidative stress in the central nervous system, thereby potentially improving autism symptoms. In this study, histopathological and biochemical alterations, as well as autism-like behaviors, were examined in the VPA-induced rat model of autism. The effects of the MOTS-c peptide on these changes were investigated to elucidate potential pathophysiological mechanisms. Thus, this study aimed to provide a basis for future research evaluating MOTS-c as a potential therapeutic agent for ASD, for which no curative treatment is currently available.

## Materials and Methods

### Animals

In this study, 32 female and 32 male offspring rats, derived from 9 female Sprague-Dawley rats (RRID: RRRC_00239), were used. The animals were obtained from the Bezmialem Vakıf University Experimental Animals Laboratory (BEDEHAL) and housed under controlled laboratory conditions (23 ± 2 °C, 55–60% humidity, 12-h light/dark cycle) in standard polypropylene cages (40 × 25 × 19 cm) with ad libitum access to food and water.

### Chemicals

VPA (Cayman Chemical, USA) was prepared at a concentration of 150 mg/mL in 0.9% saline. Rat MOTS-c (Met-Lys-Arg-Lys-Glu-Met-Gly-Tyr-Ile-Phe-Phe-Ser-Gln-Arg-Thr-Leu-Arg-Asn-Pro-Leu, 95.16% purity) (ABclonal, South Korea) was prepared at a concentration of 0.25 mg/mL in 0.9% saline. Phosphate-buffered saline (BioShop, Canada) was prepared by dissolving one tablet in 100 mL of distilled water (pH 7.4 ± 0.05).

### Induction of the Autism Model

**Fig. 1:**
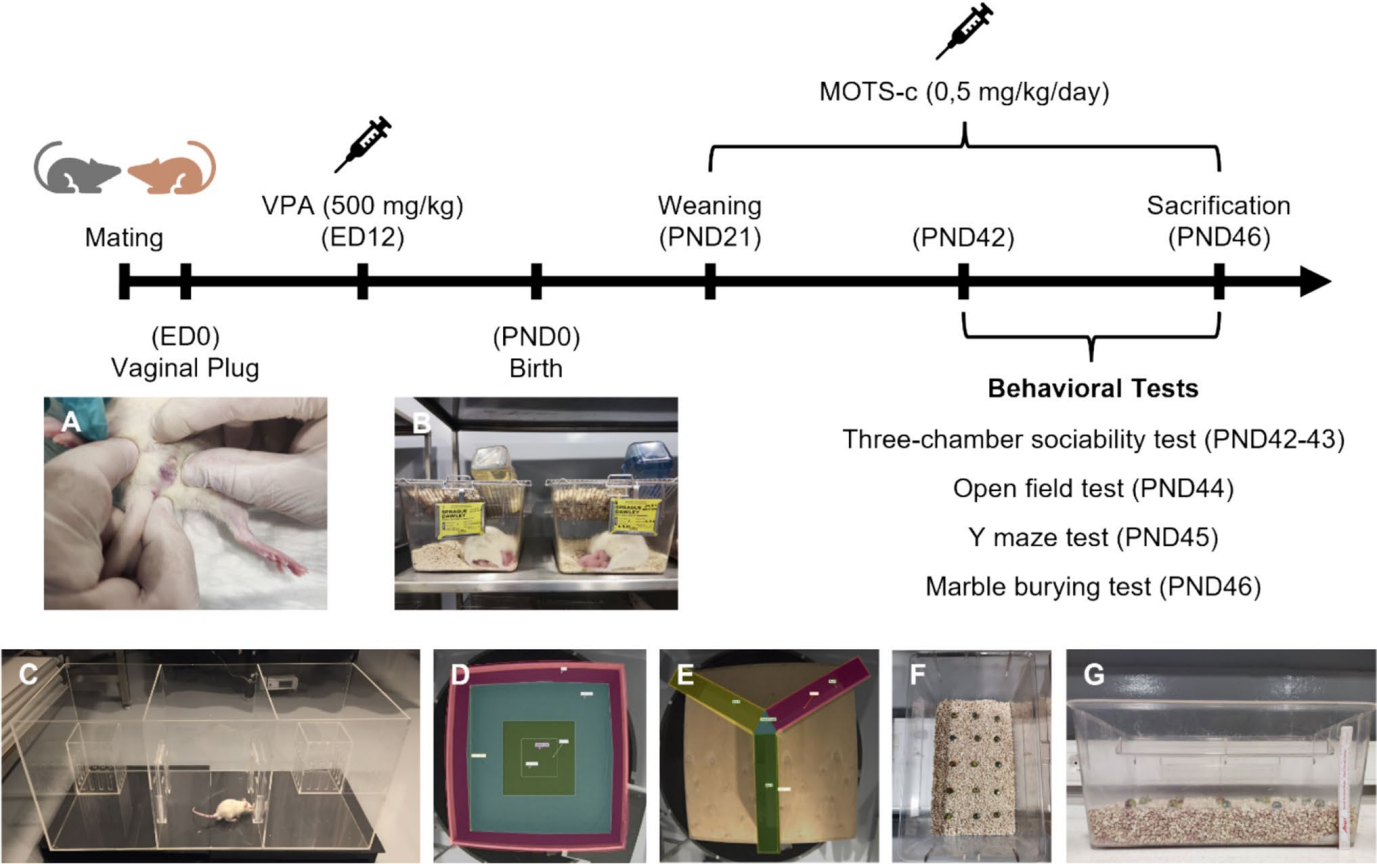
Schematic timeline illustrating the experimental design and procedures conducted throughout the study. **A** Vaginal plug examination. **B** Dams and offspring in the home cage. **C** Three-chamber sociability test apparatus. **D** Open field test apparatus. **E** Y maze test apparatus. **F** and **G** Initial setup of the marble burying test. The images show the arrangement of the 15 marbles evenly spaced on the surface of the 5 cm bedding layer at the beginning of the test. ED, embryonic day; PND, postnatal day; VPA, valproic acid

Adult male and female Sprague-Dawley rats were allowed to mate overnight at a 1:1 ratio. The following morning, pregnancy was confirmed by the presence of a vaginal plug, and 17 dams were housed individually, with this day recorded as embryonic day (ED) 0. On ED12, thirteen dams received an intraperitoneal (i.p.) injection of VPA (500 mg/kg), while four dams received an i.p. injection of the equivalent volume of 0.9% saline (3.33 mL/kg). During follow-up, six out of the 13 VPA-injected dams delivered 27 male and 21 female pups, while three out of the 4 saline-injected dams delivered 20 male and 14 female pups. The day of birth was recorded as postnatal day (PND) 0. On PND21, the litters were weaned after sex determination. Eighteen male and 18 female pups from the VPA group, and 14 male and 14 female pups from the saline group, were randomly selected for the study (Fig. 1) [25, 40, 41].

The most commonly preferred VPA doses in studies utilizing the VPA-induced autism model are 500 and 600 mg/kg [12]. A study comparing these two VPA doses demonstrated that a dose of 500 mg/kg is sufficient to induce an autism model, and the total fetal resorption rate was lower in the 500 mg/kg group [17].

Various studies have investigated the timing of VPA administration. A study comparing prenatal and postnatal VPA exposure revealed that the core symptoms of autism and significant histopathological alterations were more prominent in the prenatal model than in the postnatal model. Furthermore, the postnatal mortality rate was considerably lower in the prenatal group (4.3%) than in the postnatal group (22.7%) [10]. Another study compared the effects of VPA injections administered at different EDs and suggested that ED12, corresponding to the period of neural tube closure in rats, is the most appropriate timing to elicit behavioral alterations [42].

Most studies using the VPA-induced autism model have focused exclusively on males, with only a limited number investigating sex-based phenotypic differences [43]. Although ASD is more prevalent in males, prenatal VPA exposure has been suggested to eliminate sex differences in both animal models and humans [12]. In our study, both male and female offspring were included and analyzed as distinct experimental groups to allow descriptive comparisons across groups stratified by sex.

### Experimental Groups

**Fig. 2:**
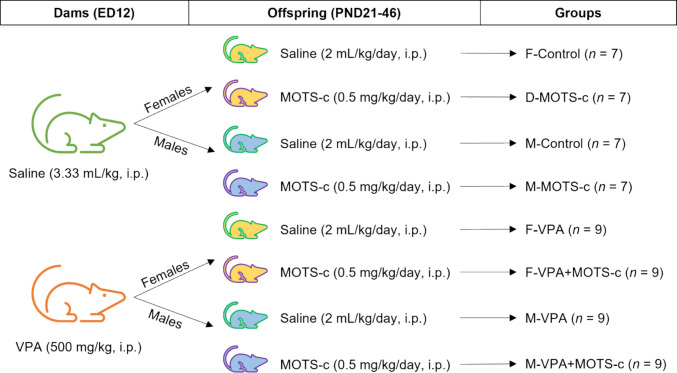
Schematic representation of the experimental groups. On embryonic day 12 (ED12), dams received either an intraperitoneal (i.p.) injection of 0.9% saline (3.33 mL/kg) or valproic acid (VPA; 500 mg/kg). Male (M) and female (F) offspring from each prenatal injection group were further divided into two postnatal treatment groups. They received either daily saline (2 mL/kg, i.p.) or MOTS-c (0.5 mg/kg, i.p.) injections between postnatal days (PND) 21 and 46. This experimental design resulted in a total of eight groups. Group names and sample sizes are indicated in the figure

All offspring were divided into two groups on the basis of prenatal VPA exposure and sex at PND21, resulting in eight experimental groups (Fig. 2) [18, 40].

- *F-Control*: Female offspring born to dams that received a single i.p. injection of 0.9% saline (3.33 mL/kg) on ED12. These rats received daily i.p. injections of 0.9% saline (2 mL/kg) from PND21 to PND46 (*n* = 7).
- *F-VPA*: Female offspring born to dams that received a single i.p. injection of VPA (500 mg/kg) on ED12. These rats received daily i.p. injections of 0.9% saline (2 mL/kg) from PND21 to PND46 (*n* = 9).
- *F-MOTS-c*: Female offspring born to dams that received a single i.p. injection of 0.9% saline (3.33 mL/kg) on ED12. These rats received daily i.p. injections of MOTS-c (0.5 mg/kg) from PND21 to PND46 (*n* = 7).
- *F-VPA*+*MOTS-c*: Female offspring born to dams that received a single i.p. injection of VPA (500 mg/kg) on ED12. These rats received daily i.p. injections of MOTS-c (0.5 mg/kg) from PND21 to PND46 (*n* = 9).
- *M-Control*: Male offspring born to dams that received a single i.p. injection of 0.9% saline (3.33 mL/kg) on ED12. These rats received daily i.p. injections of 0.9% saline (2 mL/kg) from PND21 to PND46 (*n* = 7).
- *M-VPA*: Male offspring born to dams that received a single i.p. injection of VPA (500 mg/kg) on ED12. These rats received daily i.p. injections of 0.9% saline (2 mL/kg) from PND21 to PND46 (*n* = 9).
- *M-MOTS-c*: Male offspring born to dams that received a single i.p. injection of 0.9% saline (3.33 mL/kg) on ED12. These rats received daily i.p. injections of MOTS-c (0.5 mg/kg) from PND21 to PND46 (*n* = 7).
- *M-VPA*+*MOTS-c*: Male offspring born to dams that received a single i.p. injection of VPA (500 mg/kg) on ED12. These rats received daily i.p. injections of MOTS-c (0.5 mg/kg) from PND21 to PND46 (*n* = 9).

Puberty in rats is generally considered to begin around PND28 and to extend until approximately PND56 [44, 45]. Therefore, the data collected in this study are representative of adolescent rats.

### Behavioral Tests

All rats were subjected to behavioral tests between PND42 and PND46 during the light phase of the day. The animals were acclimated to the testing room for at least 30 min before each experiment. For all tests, after each animal, the apparatus was cleaned with a 50% ethanol solution.

*Three-chamber sociability test*: The apparatus used for the three-chamber sociability test was made of plexiglass and consisted of three adjacent chambers, each measuring 30 × 30 × 40 cm (Fig. 1C). Two small identical cages (15 × 15 × 20 cm) were placed in the side chambers. The chambers were interconnected, permitting free movement between them, and the passages could be closed during specific phases of the experiment. The test consisted of three phases. The habituation phase (phase 1) was conducted on PND42, during which the test rat was placed in the central chamber with the passages closed for 5 min to allow acclimation to the apparatus. The second and third phases were conducted on PND43. In the sociability phase (phase 2), the cage in the left chamber was left empty (object), while the cage in the right chamber contained a stranger rat (stranger-1, S1). In the social novelty phase (phase 3), a novel stranger rat (stranger-2, S2) was placed in the previously empty cage. In both the second and third phases, the test rat was initially placed in the central chamber, the passages between the chambers were opened, and the rat’s behavior was recorded for 10 min. Both S1 and S2 were age- and sex-matched to the test rat and had no prior interactions with it. The recorded data were analyzed via MPC-HC (Media Player Classic-Home Cinema, v2.0.0), and the time spent by the test rat in each chamber and exploring the cages was determined. A chamber transition was defined as the test rat crossing into a chamber with all four paws.

On the basis of the collected data, the Sociability Index (SI) and Social Novelty Index (SNI) were calculated via the following formulas:

$$SI \left(\%\right)=\frac{Time spent in the S1 chamber-Time spent in the object chamber}{Time spent in the S1 chamber+Time spent in the object chamber}\times 100$$

$$SNI \left(\%\right)=\frac{Time spent in the S2 chamber-Time spent in the S1 chamber}{Time spent in the S2 chamber+Time spent in the S1 chamber}\times 100$$

Since the social novelty phase is used to assess social memory and preference for social novelty, rats meeting the following criteria were excluded from both the analyses: (1) did not visit both chambers during the sociability phase and (2) did not interact with either S1 or S2 during the social novelty phase [46].

*Open field test*: The open field apparatus consisted of a 100 × 100 × 20 cm box made of plywood (Fig. 1D). The open field test was conducted on PND44. At the start of the experiment, the test rat was placed at the center of the box and allowed to explore freely for 5 min. The entire session was recorded via EthoVision XT software (v11.5; RRID: SCR_000441). During the test, the following parameters were analyzed: the total distance traveled, immobility duration, and time spent in the inner zone (a 50 × 50 cm area marked at the center of the apparatus) and the outer zone (the remaining area). These parameters were determined on the basis of the movement of the rat’s body center, as tracked by the software. Additionally, the activity status was assessed via pixel-based motion analysis in the software.

*Y maze test*: The Y maze apparatus was made of plexiglass and consisted of three identical arms (10 × 50 × 20 cm) positioned at a 120° angle to each other (Fig. 1E). The test was conducted on PND45. At the beginning of the experiment, each rat was placed in a predetermined arm. The rats were allowed to explore freely, and their movements were recorded for 8 min via EthoVision XT (v11.5) software. Maximum alternations (total arm entries − (the number of arms − 1)) and alternation counts (the number of consecutive entries into three different arms) were determined on the basis of the movement of the rat’s body center, as tracked by the software. The percentage of spontaneous alternation (SA) was calculated according to the following formula [47]:

$$SA \left(\%\right)=\frac{\left(Alternation counts\right)}{(Maximum alternations)}\times 100$$

*Marble burying test*: The marble burying test was conducted in standard cages where the rats were housed to minimize neophobia. The test was conducted on PND46. A 5 cm layer of clean corncob bedding was placed on the cage floor, and fifteen glass marbles (15 mm in diameter) were arranged in a 5 × 3 grid and evenly spaced from each other and the cage walls (Fig. 1F and G). Each rat was tested individually and allowed to explore freely for 20 min. At the end of the test, the number of buried marbles was counted by three researchers. Marbles with at least half of their surface covered were considered buried [10].

### Tissue Collection

Following the completion of the behavioral tests, the rats were deeply anesthetized on PND46 via i.p. injection of ketamine-xylazine (80–10 mg/kg). The depth of anesthesia was assessed by the absence of reflexive responses to painful stimuli. Intracardiac blood was collected for biochemical analyses, and the rats were decapitated for brain dissection. Blood samples were collected into ethylenediaminetetraacetic acid–containing tubes and kept at +4 °C for approximately 30 min. The samples were then centrifuged at 3000 RPM for 10 min. The plasma samples were separated into Eppendorf tubes and stored at −80 °C until biochemical analyses were performed.

Following decapitation, the overlying skin and muscle tissues of the skull were removed. A posterior incision was made in the skull via bone scissors, and the occipital bone over the cerebellum was carefully lifted. The parietal bones were cut from the posterolateral to the anterolateral corners, ensuring that the cerebral tissue remained intact while the bone was carefully removed. The residual bone tissue surrounding the cerebrum was also removed. The neural connections were gently separated via a spatula, and the cerebrum and cerebellum were extracted and placed in 0.9% saline. The brainstem was then separated by making a coronal incision at the posterior boundary of the cerebellum. The brain was subsequently placed in a brain matrix. The cerebrum and cerebellum were separated via a coronal incision. Both the cerebrum and the cerebellum were subsequently divided into two hemispheres via a midsagittal incision. The left cerebral and cerebellar hemispheres were placed in 10% neutral buffered formalin for histological analysis. In the right cerebral hemisphere, the corpus callosum was identified along the midsagittal plane, and the frontal section was separated by a coronal incision at the anterior boundary of the genu of the corpus callosum. Noncortical structures were removed from this section to isolate the PFC [48]. The dissected PFC tissues were immediately wrapped in aluminum foil, frozen in liquid nitrogen, and stored at –80 °C for biochemical analyses.

### Histopathological Examinations

**Fig. 3:**
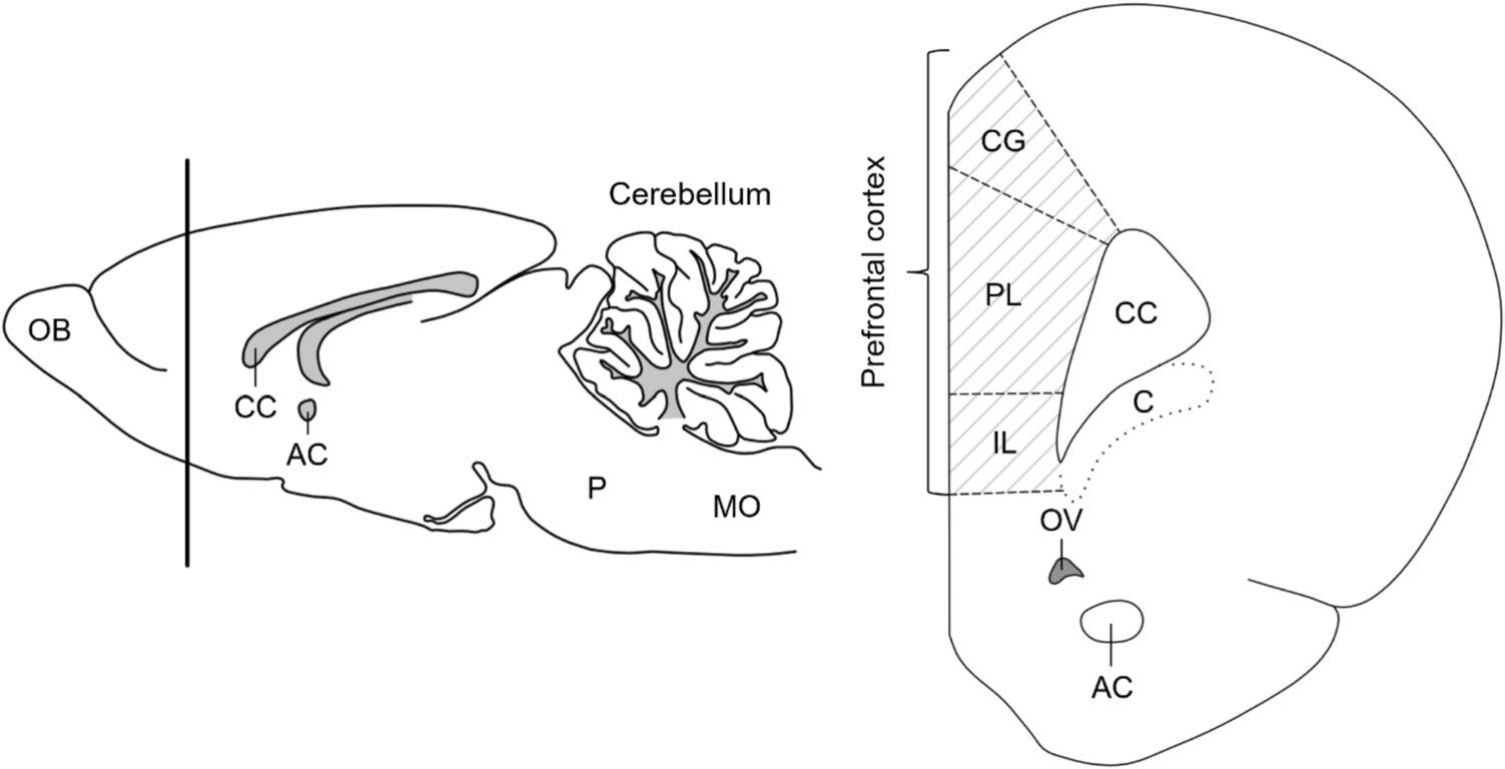
Schematic representation of the histological section. AC, anterior commissure; C, claustrum; CC, corpus callosum; CG, cingulate cortex; IL, infralimbic cortex; MO, medulla oblongata; OB, olfactory bulb; OV, olfactory ventricle; P, pons; PL, prelimbic cortex. The figure was adapted and redrawn on the basis of illustrations from “The Rat Brain in Stereotaxic Coordinates” [50]

The cerebral and cerebellar tissues, which were fixed overnight in a 10% neutral buffered formalin solution, were dehydrated by passing through an ascending series of ethanol concentrations. The tissues were then cleared using toluene. The tissues were subsequently embedded in paraffin after being placed in a toluene-paraffin mixture and pure paraffin. Serial 5-µm-thick sections were obtained in the coronal plane from the anterior cerebrum and in the sagittal plane from the cerebellum from paraffin-embedded tissue blocks. One section was collected, and five consecutive sections were discarded. Three serial sections of the anterior cerebrum were selected for staining to study the PFC by examining the sections under a light microscope (Fig. 3). Before histological staining, the paraffin in the sections was removed with toluene, and the tissues were passed through a descending ethanol series, followed by immersion in distilled water for rehydration. The rehydrated sections were incubated for 10 min in 0.1% cresyl violet solution (pH 3, 37 °C). The tissues were then washed with distilled water and differentiated in 95% ethanol. The tissues were subsequently passed through 100% ethanol for dehydration, cleared with toluene, and mounted with Entellan. The sections from each cerebrum were examined for histopathological assessment of the PFC. Neuronal damage was evaluated on the basis of the presence of one or more of the following findings: hyperchromatic and shrunken nuclei, cytoplasmic eosinophilia/basophilia, and karyorrhectic nuclei. Histopathological scoring was performed by assessing the proportion of damaged neurons in the tissue (0: no damage, 4: diffuse damage, with a scale from 0 to 4) (Table 1) [49].

**Table 1:** Histopathological scores

Criteria	Score
Normal: No damage or rare isolated apoptotic neurons	0
Rare neuronal damage: < 5 clusters	1
Intermittent neuronal damage: 5–15 clusters	2
Frequent neuronal damage: > 15 clusters	3
Diffuse neuronal damage	4

For each cerebellum, three serial sections were stained with cresyl violet via the same staining protocol and subsequently examined. Three random areas containing the molecular layer from each section were imaged under a light microscope (Olympus BX61, DP72, Japan; RRID: SCR_020343) at ×40 magnification. In the obtained micrographs, Purkinje cells were counted. The transition zone between the molecular and granular layers parallel to the Purkinje cells was measured in millimeters via cellSens Standard software (Olympus, Japan; RRID: SCR_014551). The number of Purkinje cells per millimeter was then calculated [51].

### Biochemical Parameters

Rat BDNF Enzyme-Linked Immunosorbent Assay (ELISA) Kit (Sunred Biological Technology, China; 201-11-0477), Rat BH4 ELISA Kit (Sunred Biological Technology, China; 201-11-1723), Malondialdehyde (MDA) Colorimetric Assay Kit (Thiobarbituric Acid (TBA) Method) (Elabscience, USA; E-BC-K025-M), Total Superoxide Dismutase (T-SOD) Activity Assay Kit (Hydroxylamine Method) (Elabscience, USA; E-BC-K019-M), and Total Glutathione (T-GSH)/Oxidized Glutathione (GSSG) Colorimetric Assay Kit (Elabscience, USA; E-BC-K097-M) were used.

Tissue homogenates were prepared at a 20% (w/v) concentration by adding phosphate-buffered saline to PFC tissue samples placed in Eppendorf tubes. Stainless steel beads (0.9–2.0 mm in diameter) were added, and the samples were homogenized for 5 min at maximum speed in a Bullet Blender Tissue Homogenizer (Next Advance, USA). The homogenates were centrifuged at 5000 g for 10 min, and the supernatants were carefully separated via a micropipette. MDA levels, T-SOD activity, and T-GSH levels were measured in the supernatants via commercially available colorimetric assay kits [52]. BH4 and BDNF levels were measured in the plasma samples via ELISA kits [53]. For all tests, the protocols of the commercial kits were strictly followed. Optical densities were determined using a microplate reader with KCjunior software (Bio-Tek Instruments, USA) at wavelengths of 532 nm for MDA, 550 nm for T-SOD activity, 412 nm for T-GSH levels, and 450 nm for BH4 and BDNF ELISA kits.

### Statistical Analysis

On the basis of the “social index” comparison values, which maximize the sample size, a minimum of *n* = 7 animals per group would be required to detect an expected maximum mean difference of 1.5 units (with a standard deviation of 0.7 units) between the groups, with 95% confidence (1–α) and 80% power (1–β) (G*Power 3.1, effect size = 0.56) [15]. Considering the potential risk of postnatal mortality and possible exclusions during the behavioral experiments, a total of 64 animals were included in the study, with *n* = 9 animals in the VPA groups.

Statistical analyses were performed via IBM SPSS (Statistical Package for the Social Sciences) software (RRID: SCR_016479) version 29. The normality of the dependent variables was assessed via analytical tests (Kolmogorov-Smirnov and Shapiro-Wilk) and visually inspected via histograms. Graphs were generated via GraphPad Prism software version 8. All the data were visualized via box-and-whisker plots displaying individual data points, medians, interquartile ranges, and minimum-to-maximum values (whiskers), regardless of the statistical test applied, to ensure consistency and clarity in representing the data distribution. One-way analysis of variance (ANOVA) test was used to compare multiple groups when all groups followed a normal distribution. When a significant difference was detected, post hoc analyses were performed via the Bonferroni test for multiple comparisons. Homogeneity of variances was assessed with the Levene test. For comparisons of multiple groups that did not meet normality assumptions, the Kruskal-Wallis test was used, followed by pairwise comparisons automatically computed by software with Bonferroni correction. For within-group comparisons of paired measurements, the paired-sample *t*-test was applied for normally distributed differences, whereas the Wilcoxon signed-rank test was applied for groups that did not meet normality assumptions. For all analyses, *p* < 0.05 was considered statistically significant.

## Results

### Dams and Offspring

**Fig. 4:**
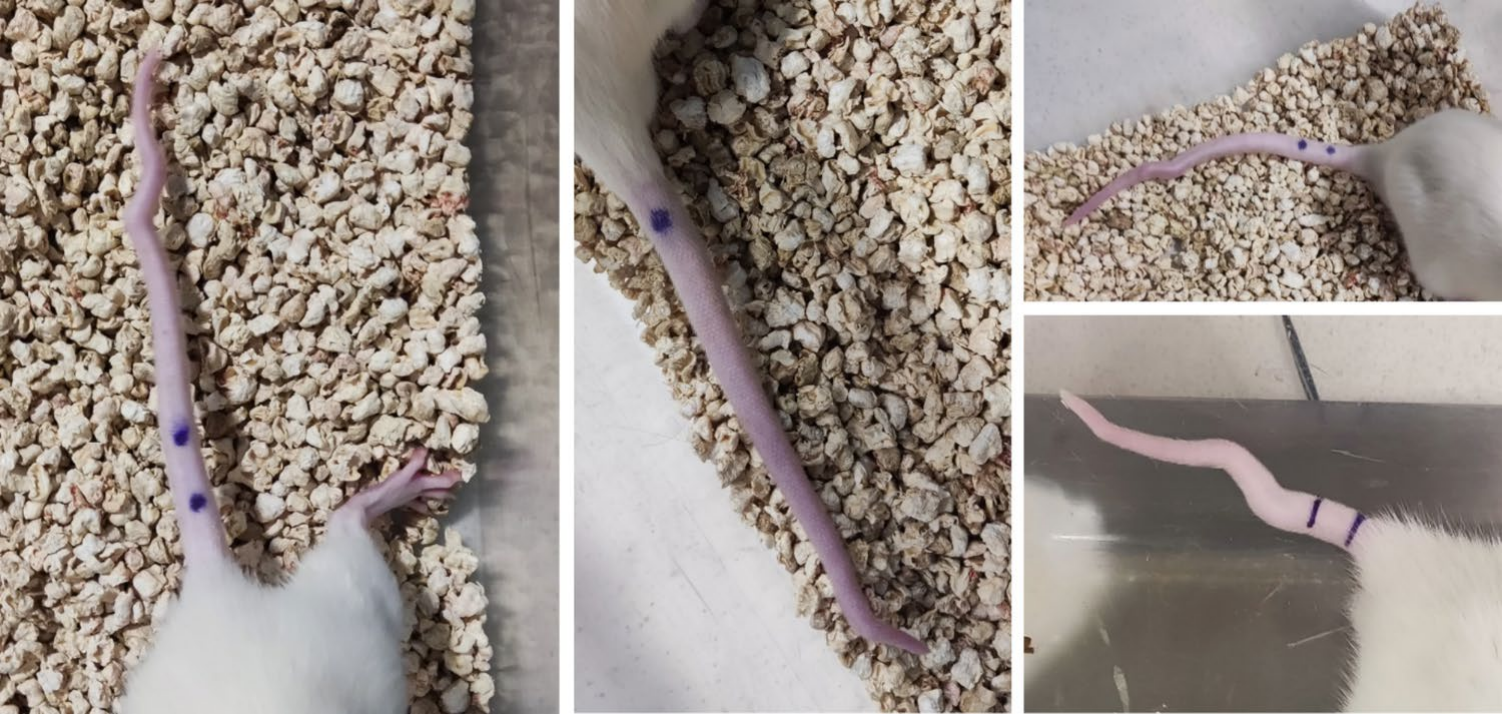
Representative images showing tail-kink deformities observed in offspring prenatally exposed to valproic acid (VPA). Tail malformations were observed at different segments of the tail

Total fetal reabsorption was observed in 7 out of 13 dams (53.8%) injected with VPA on ED12, while the remaining 6 dams gave birth to a mean of 6 pups each. In the saline-injected group, total fetal reabsorption occurred in 1 out of 4 dams (25%), whereas the remaining 3 dams gave birth to a mean of 11.33 pups each. Among the 48 offspring born to VPA-injected dams, tail kinks at different levels were observed in 20 pups (41.66%) (Fig. 4). In rodents, total fetal reabsorption and tail-kink deformities associated with the teratogenic effects of VPA have been reported [10, 17, 54, 55].

### Behavioral Tests

#### Three-Chamber Sociability Test

**Fig. 5:**
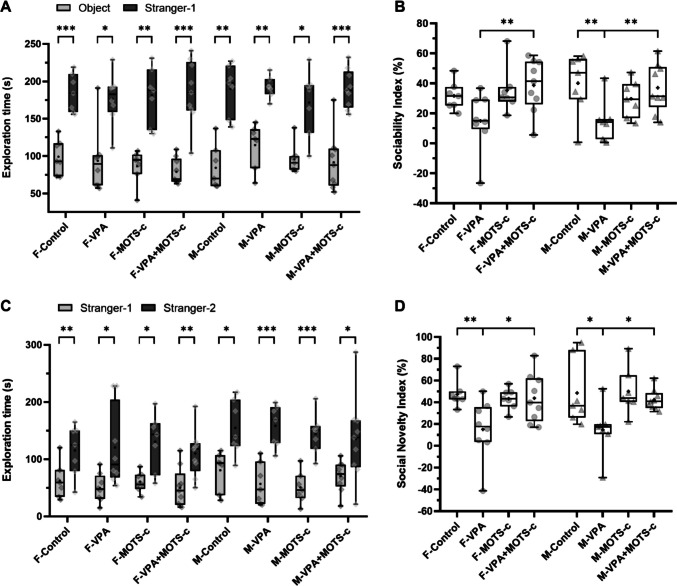
Three-chamber sociability test assessing social interaction and social novelty preference (*n*_*F-Control*_ = 7, *n*_*F-VPA*_ = 8, *n*_*F-MOTS-c*_ = 7, *n*_*F-VPA*+*MOTS-c*_ = 9, *n*_*M-Control*_ = 7, *n*_*M-VPA*_ = 7, *n*_*M-MOTS-c*_ = 7, *n*_*M-VPA*+*MOTS-c*_ = 9; animals meeting the exclusion criteria described in detail in the Methods section were excluded from both analyses). **A**, **B** Sociability phase, exploration time (**A**) and sociability index (**B**). **C**, **D** Social novelty phase, exploration time (**C**) and social novelty index (**D**). F, female; M, male; VPA, valproic acid. *p <.05, **p <.01, ***p <.001

In the three-chamber sociability test, during the sociability phase, no statistically significant differences were found between the groups in terms of the time spent exploring the object and the S1 cage (Kruskal-Wallis, *H*(7) = 7.910, *p* = 0.341; one-way ANOVA, *F*(7) = 0.327, *p* = 0.938; respectively). When the exploration times of the object and the S1 cage were compared within each group, the time spent exploring the S1 cage was significantly greater than the time spent exploring the object (paired-sample *t*-test, F-Control: *T*(6) = 7.555, *p* < 0.001; F-VPA: *T*(7) = 3.193, *p* = 0.015; F-MOTS-c: *T*(6) = 5.695, *p* = 0.001; F-VPA+MOTS-c: *T*(8) = 5.832, *p* < 0.001; M-Control: *T*(6) = 4.853, *p* = 0.003; M-VPA: *T*(6) = 5.090, *p* = 0.002; M-MOTS-c: *T*(6) = 4.406, *p* = 0.005; M-VPA+MOTS-c: *T*(8) = 7.914, *p* < 0.001) (Fig. 5A). A statistically significant difference was found between the groups for the SI values (Kruskal-Wallis, *H*(7) = 17.921, *p* = 0.012). SI was lower in the M-VPA group than in the M-Control group (*p* = 0.004), whereas the difference observed in the F-VPA group compared with the F-Control group was not statistically significant (*p* = 0.083). SI was higher in the F-VPA+MOTS-c (*p* = 0.008) and M-VPA+MOTS-c (*p* = 0.008) groups than in their respective VPA groups (Fig. 5B).

During the social novelty phase, no statistically significant differences were found between the groups in terms of the time spent exploring the S1 cage and the S2 cage (Kruskal-Wallis, *H*(7) = 6.957, *p* = 0.433; *H*(7) = 7.628, *p* = 0.366, respectively). Within each group, the time spent exploring the S2 cage was significantly greater than that spent exploring the S1 cage (Wilcoxon signed-rank test, F-VPA+MOTS-c: *Z* = 2.666, *p* = 0.008; paired-sample *t*-test, F-Control: *T*(6) = 5.082, *p* = 0.002; F-VPA: *T*(7) = 2.684, *p* = 0.031; F-MOTS-c: *T*(6) = 3.125, *p* = 0.020; M-Control: *T*(6) = 3.275, *p* = 0.017; M-VPA: *T*(6) = 6.603, *p* < 0.001; M-MOTS-c: *T*(6) = 6.496, *p* < 0.001; M-VPA+MOTS-c: *T*(8) = 2.786, *p* = 0.024) (Fig. 5C). A statistically significant difference in SNI values was found between the groups (Kruskal–Wallis, *H*(7) = 18.176, *p* = 0.011). SNI was lower in the F-VPA group than in the F-Control group (*p* = 0.006) and in the M-VPA group than in the M-Control group (*p* = 0.044). SNI was higher in the F-VPA+MOTS-c group than in the F-VPA group (*p* = 0.033) and in the M-VPA+MOTS-c group than in the M-VPA group (*p* = 0.021) (Fig. 5D).

#### Y Maze Test

**Fig. 6:**
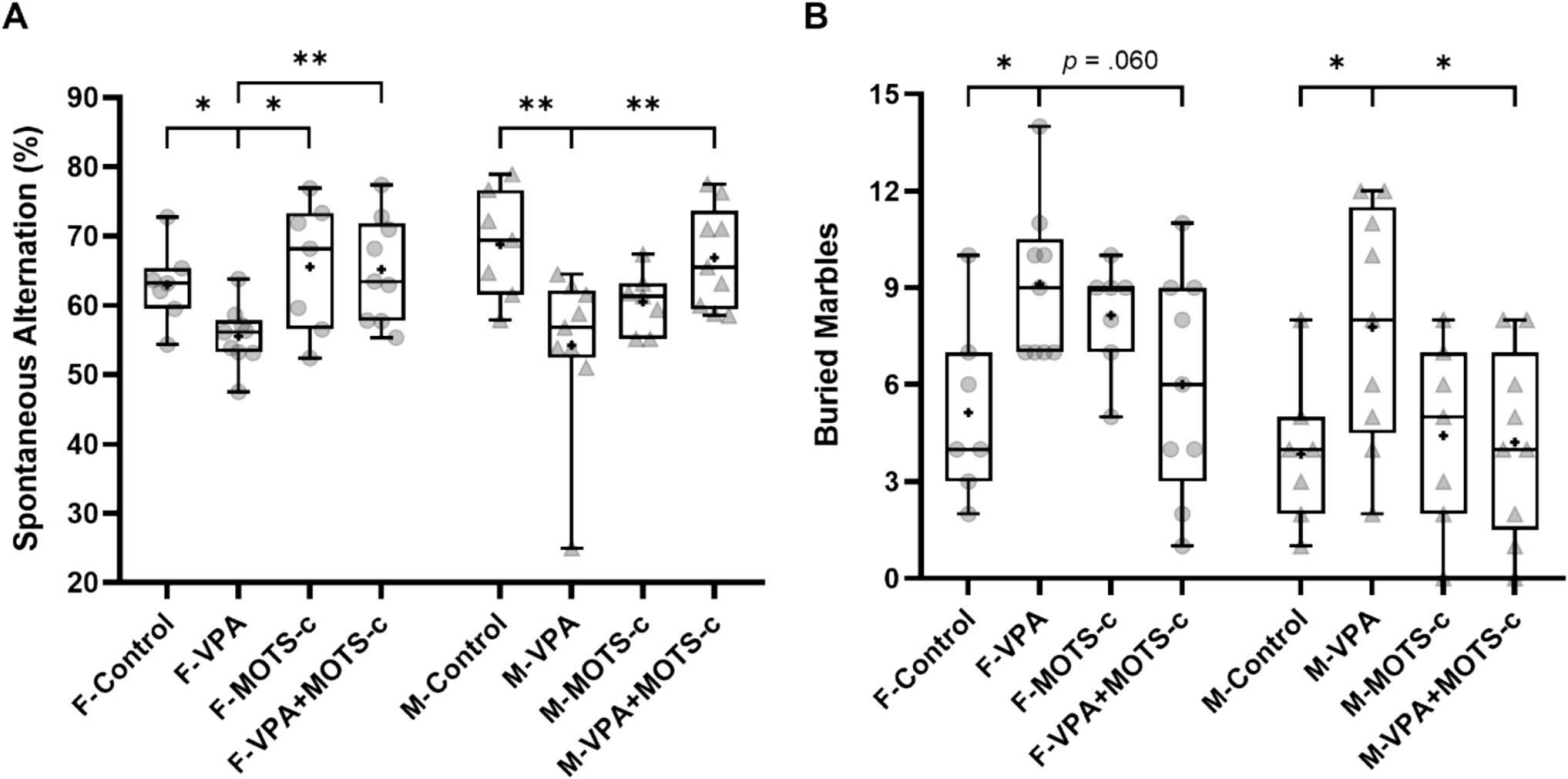
Behavioral assessments of repetitive behaviors (*n*_*F-Control*_ = 7, *n*_*F-VPA*_ = 9, *n*_*F-MOTS-c*_ = 7, *n*_*F-VPA*+*MOTS-c*_ = 9, *n*_*M-Control*_ = 7, *n*_*M-VPA*_ = 9, *n*_*M-MOTS-c*_ = 7, *n*_*M-VPA*+*MOTS-c*_ = 9). **A** Spontaneous alternation percentage in the Y maze test. **B** Number of marbles buried in the marble burying test. F, female; M, male; VPA, valproic acid. *p <.05, **p <.01, ***p <.001

In the Y maze test, the difference in SA was statistically significant among the groups (Kruskal-Wallis, *H*(7) = 22.003, *p* = 0.003). SA was lower in the F-VPA group than in the F-Control group (*p* = 0.024) and in the M-VPA group than in the M-Control group (*p* = 0.003). SA was higher in the F-VPA+MOTS-c group than in the F-VPA group (*p* = 0.007) and in the M-VPA+MOTS-c group than in the M-VPA group (*p* = 0.006) (Fig. 6A).

#### Marble Burying Test

In the marble burying test, the difference in the number of buried marbles was statistically significant among the groups (Kruskal-Wallis, *H*(7) = 20.858, *p* = 0.004). The number of buried marbles was greater in the F-VPA group than in the F-Control group (*p* = 0.023) and in the M-VPA group than in the M-Control group (*p* = 0.022). The number of buried marbles was lower in the M-VPA+MOTS-c group than in the M-VPA group (*p* = 0.028), whereas the difference observed in the F-VPA group compared with the F-VPA+MOTS-c group was not statistically significant (*p* = 0.060) (Fig. 6B).

#### Open Field Test

**Fig. 7:**
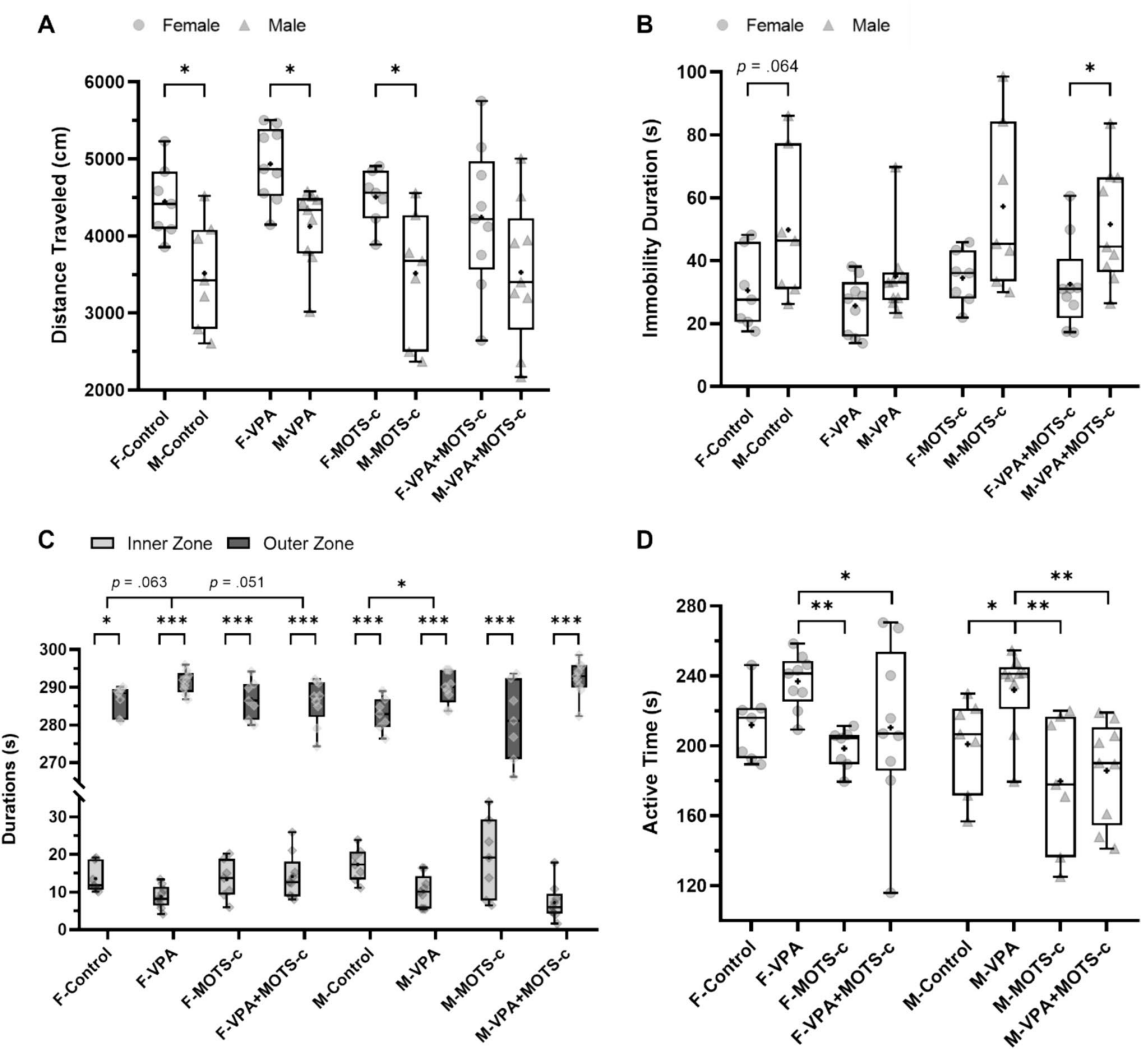
Open field test assessing locomotor activity and anxiety-like behavior (*n*_*F-Control*_ = 7, *n*_*F-VPA*_ = 9, *n*_*F-MOTS-c*_ = 7, *n*_*F-VPA*+*MOTS-c*_ = 9, *n*_*M-Control*_ = 7, *n*_*M-VPA*_ = 9, *n*_*M-MOTS-c*_ = 7, *n*_*M-VPA*+*MOTS-c*_ = 9). **A** Total distance traveled, indicating overall locomotor activity. **B** Duration of immobility, reflecting reduced movement. **C** Time spent in the inner and outer zones as an index of anxiety-like behavior. D Active time quantified by pixel-based motion analysis. F, female; M, male; VPA, valproic acid. *p <.05, **p <.01, ***p <.001

In the open field test, a statistically significant difference was found between the groups in terms of the distance traveled (cm) (Kruskal-Wallis, *H*(7) = 24.324, *p* < 0.001). Accordingly, the F-Control group traveled a greater distance than did the M-Control group (*p* = 0.039), the F-VPA group traveled a greater distance than did the M-VPA group (*p* = 0.016), and the F-MOTS-c group traveled a greater distance than did the M-MOTS-c group (*p* = 0.017). No significant differences were found between the F-Control and F-VPA groups (*p* = 0.175) or between the M-Control and M-VPA groups (*p* = 0.194) (Fig. 7A).

A statistically significant difference was found between the groups in terms of the duration of immobility (Kruskal-Wallis, *H*(7) = 19.102, *p* = 0.008). Compared with the M-VPA+MOTS-c group, the F-VPA+MOTS-c group remained immobile for a shorter duration (*p* = 0.025). No significant differences were found between the F-VPA group and the F-Control group (*p* = 0.534) or between the M-VPA group and the M-Control group (*p* = 0.174). Similarly, no significant differences were detected between the F-VPA and F-VPA+MOTS-c groups (*p* = 0.365) or between the M-VPA and M-VPA+MOTS-c groups (*p* = 0.065) (Fig. 7B).

During the open field test, within each group, the time spent in the outer zone was significantly longer than that in the inner zone (Wilcoxon signed-rank test, F-Control: *Z* = 2.366, *p* = 0.018; paired-sample *t*-test, F-VPA: *T*(8) = 141.92, *p* < 0.001; F-MOTS-c: *T*(6) = 69.97, *p* < 0.001; F-VPA+MOTS-c: *T*(8) = 68.34, *p* < 0.001; M-Control: *T*(6) = 79.45, *p* < 0.001; M-VPA: *T*(8) = 97.70, *p* < 0.001; M-MOTS-c: *T*(6) = 33.07, *p* < 0.001; M-VPA+MOTS-c: *T*(8) = 91.05, *p* < 0.001). A statistically significant difference was found between the groups regarding the time spent in the inner zone (Kruskal-Wallis, *H*(7) = 22.621, *p* = 0.002). Accordingly, the time spent in the inner zone was shorter in the M-VPA group than in the M-Control group (*p* = 0.013), whereas the difference observed between the F-VPA and F-Control groups was not statistically significant (*p* = 0.063). No significant difference was also found between the F-VPA and F-VPA+MOTS-c groups (*p* = 0.051) or between the M-VPA and the M-VPA+MOTS-c groups (*p* = 0.232) (Fig. 7C).

A statistically significant difference was also found between the groups in terms of activity status data obtained from the pixel analysis of the software (Kruskal-Wallis, *H*(7) = 24.340, *p* < 0.001). The active time was longer in the M-VPA group than in the M-Control group (*p* = 0.048), whereas the difference observed in the F-VPA group compared with the F-Control group was not statistically significant (*p* = 0.072). The active time was shorter in the F-VPA+MOTS-c (*p* = 0.048) and M-VPA+MOTS-c (*p* = 0.002) groups than in their respective VPA groups (Fig. 7D).

### Histopathological Examinations

#### Cerebellum

**Fig. 8:**
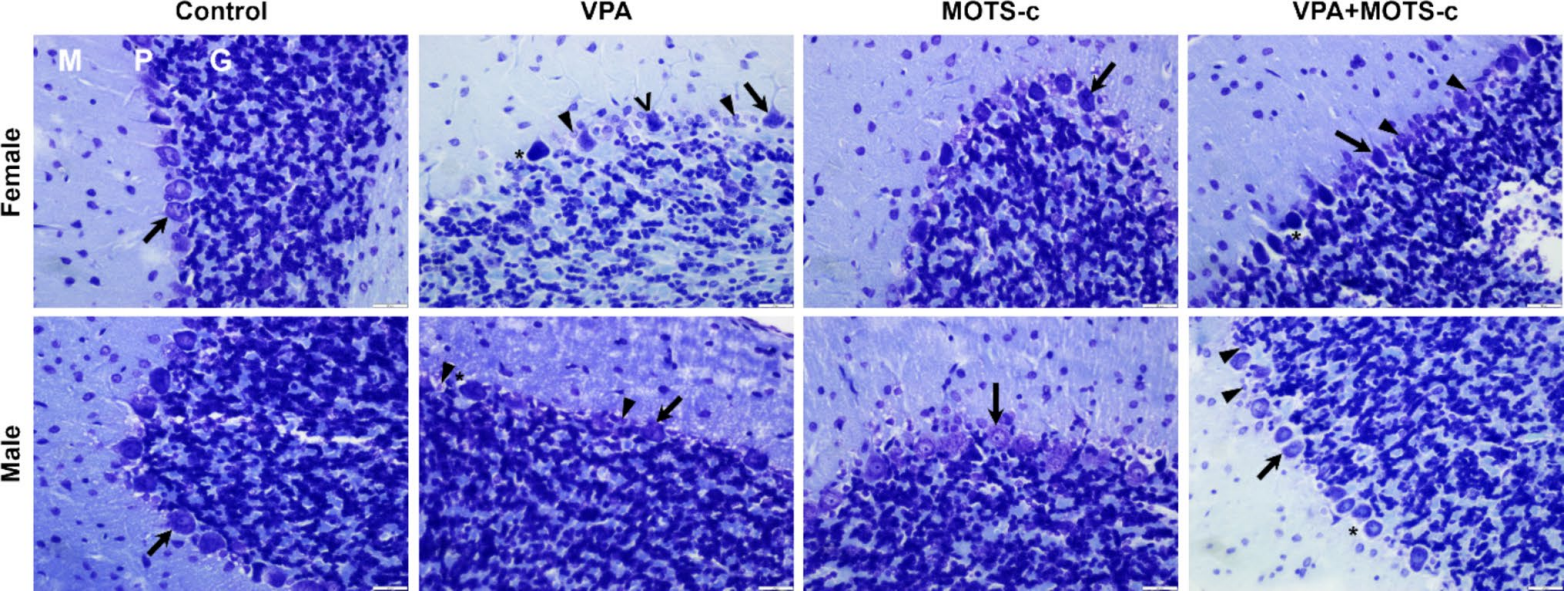
Representative cerebellar sections showing Purkinje cells (arrow), perineuronal space (*), degenerated Purkinje cells (˄), and glial cells with euchromatic nuclei (arrowhead). G, granular layer; M, molecular layer; P, Purkinje cell layer; VPA, valproic acid. Cresyl violet staining. Scale bar = 20 µm

The cerebellar cortex exhibited normal histological features in the Control groups, whereas various histopathological alterations were observed in both sexes of the VPA groups. The number of Purkinje cells in the VPA groups was reduced, and their cell bodies appeared shrunken compared with those in the Control groups. Marked degeneration was noted in Purkinje cells, characterized by prominent perineuronal spaces. Additionally, an increased number of glial cells with euchromatic nuclei surrounding damaged Purkinje cells was observed. Although histopathological findings similar to those in the VPA groups were also detected in the female and male VPA+MOTS-c groups, a noticeable reduction in Purkinje cell damage was observed in certain areas (Fig. 8).

**Fig. 9:**
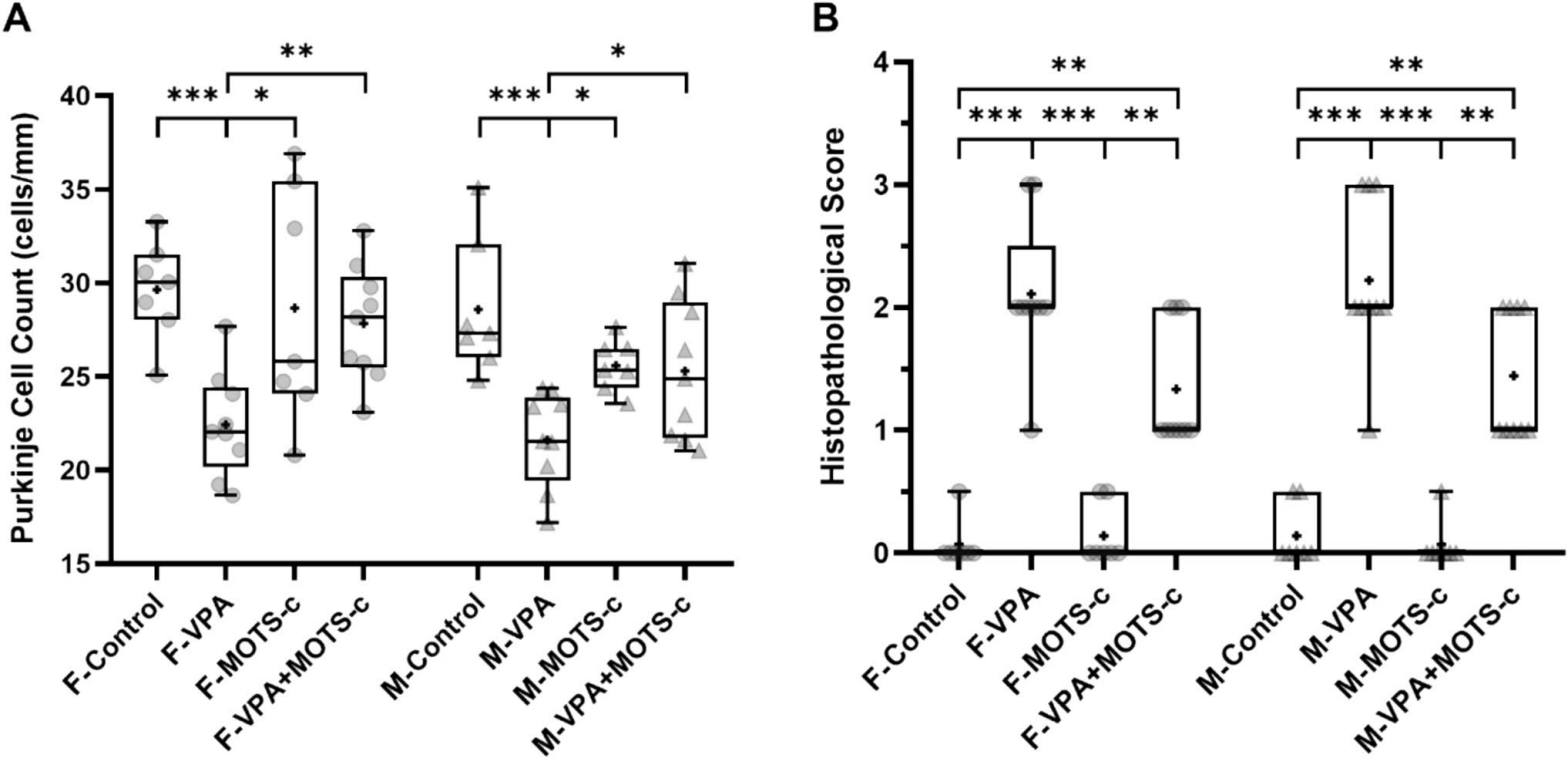
Quantitative histological analysis (*n*_*F-Control*_ = 7, *n*_*F-VPA*_ = 9, *n*_*F-MOTS-c*_ = 7, *n*_*F-VPA*+*MOTS-c*_ = 9, *n*_*M-Control*_ = 7, *n*_*M-VPA*_ = 9, *n*_*M-MOTS-c*_ = 7, *n*_*M-VPA*+*MOTS-c*_ = 9). **A** Linear density of Purkinje cells in the cerebellum (mean number per millimeter). **B** Histopathological scoring of the prefrontal cortex. F, female; M, male; VPA, valproic acid. *p <.05, **p <.01, ***p <.001

The difference in the Purkinje cell count per unit length among the groups was statistically significant (Kruskal-Wallis, *H*(7) = 29.750, *p* < 0.001). Accordingly, the Purkinje cell count was lower in the F-VPA group than in the F-Control group (*p* < 0.001) and in the M-VPA group than in the M-Control group (*p* < 0.001). The Purkinje cell count was higher in the F-VPA+MOTS-c group than in the F-VPA group (*p* = 0.003) and in the M-VPA+MOTS-c group than in the M-VPA group (*p* = 0.048) (Fig. 9A).

#### PFC

The difference in neocortical histopathological scores among the groups was statistically significant (Kruskal–Wallis, *H*(7) = 53.654, *p* < 0.001). Accordingly, the histopathological score was higher in the F-VPA group than in the F-Control group (*p* < 0.001) and in the M-VPA group than in the M-Control group (*p* < 0.001). No statistically significant differences were found between the F-VPA and F-VPA+MOTS-c groups (*p* = 0.205) or between the M-VPA and M-VPA+MOTS-c groups (*p* = 0.187). The histopathological score was also higher in the F-VPA+MOTS-c group than in the F-Control group (*p* = 0.003) and in the M-VPA+MOTS-c group than in the M-Control group (*p* = 0.004) (Fig. 9B).

**Fig. 10:**
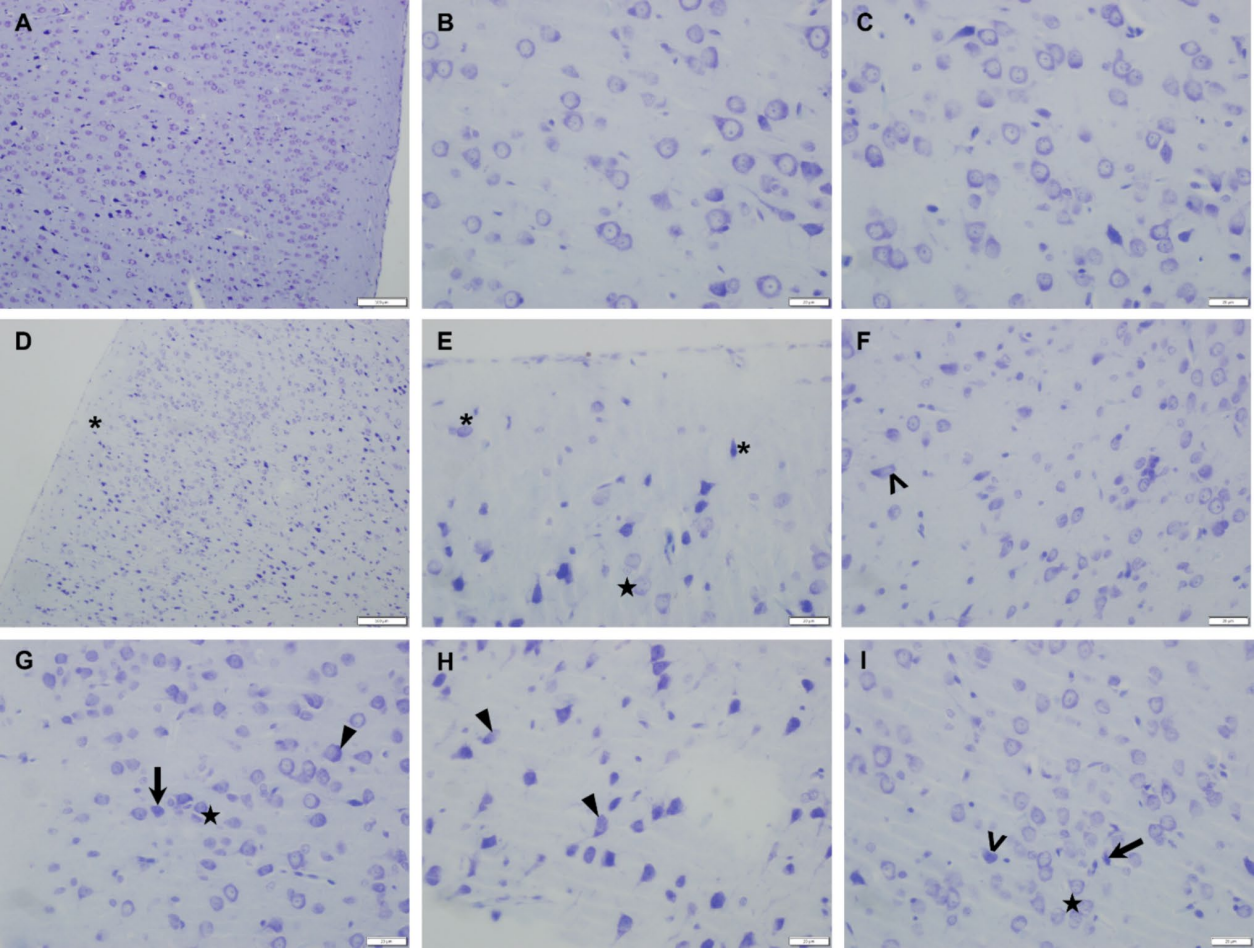
Histological evaluation of the prefrontal cortex. **A**–**C** Control groups. Neurons in the neocortex exhibit normal morphology. **D**–**I** VPA groups. Ectopically located neurons in the molecular layer (*), neurons with karyorrhectic nuclei (˄), degenerating neurons with basophilic cytoplasm (arrow), cytoplasmic vacuolization in neurons (arrowhead), and increased nucleoli in the nuclei of the granular cells (star) were observed. Cresyl violet staining. Scale bars = 100 µm (**A**, **D**), 20 µm (**B**, **C**, **E**–**I**)

In the VPA groups, numerous ectopically located neurons were observed in the molecular layer of the PFC. Additionally, compared with those in the Control groups, pyramidal neurons were widely distributed across almost all layers of the PFC (Fig. 10D and E). Moreover, more nucleoli were observed in the nuclei of granular cells in the VPA groups than in the Control groups (Fig. 10E, G, and I). Similar histopathological alterations were observed across male and female groups.

### Oxidative Stress Markers

**Fig. 11:**
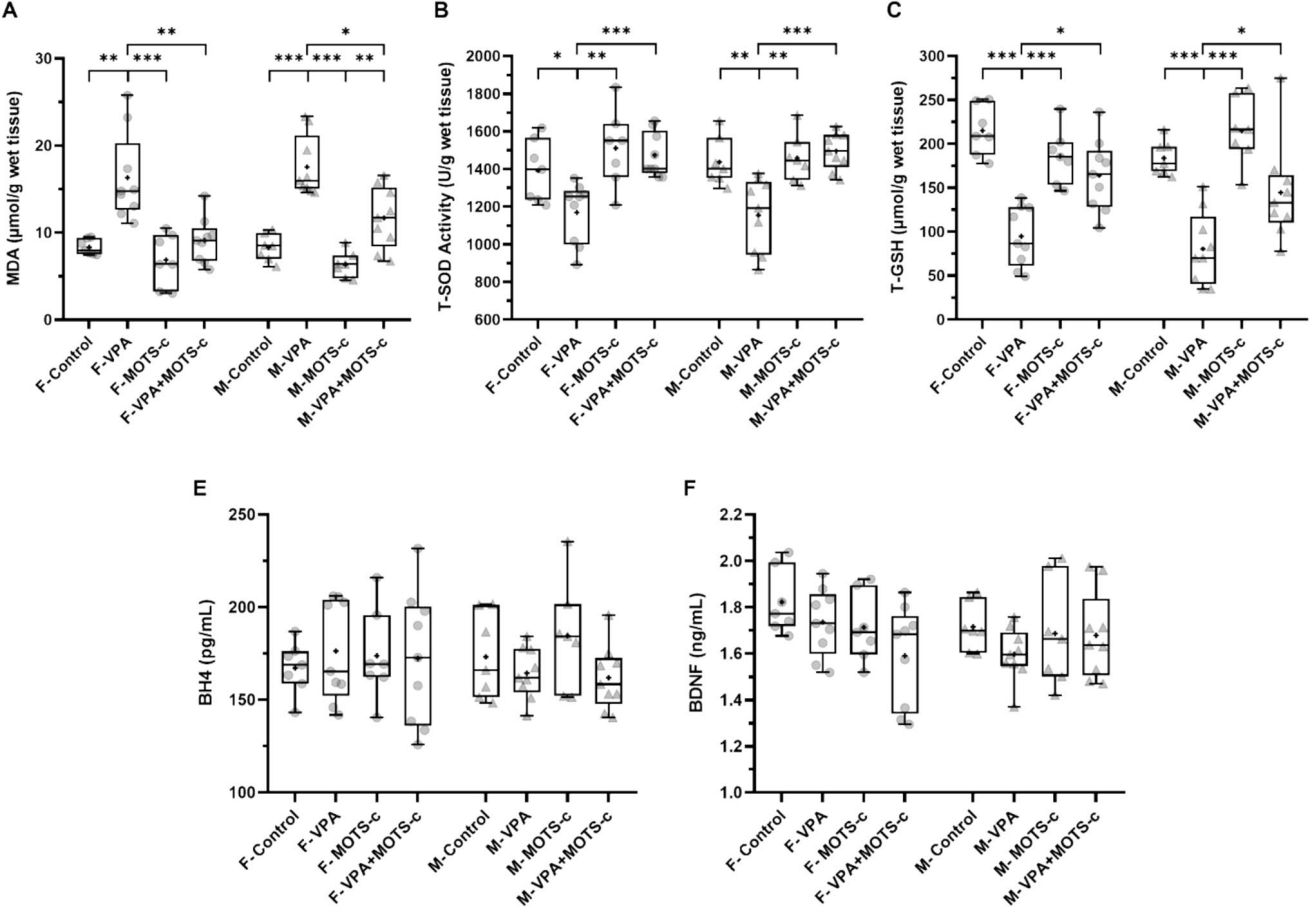
Oxidative stress parameters in the prefrontal cortex and plasma concentrations of tetrahydrobiopterin (BH4) and brain-derived neurotrophic factor (BDNF) (*n*_*F-Control*_ = 7, *n*_*F-VPA*_ = 9, *n*_*F-MOTS-c*_ = 7, *n*_*F-VPA*+*MOTS-c*_ = 9, *n*_*M-Control*_ = 7, *n*_*M-VPA*_ = 9, *n*_*M-MOTS-c*_ = 7, *n*_*M-VPA*+*MOTS-c*_ = 9). **A** Malondialdehyde (MDA) levels. **B** Total superoxide dismutase (T-SOD) activity. **C** Total glutathione (T-GSH) levels. **D** Plasma BH4 concentrations. **E** Plasma BDNF concentrations. F, female; M, male; VPA, valproic acid. *p <.05, **p <.01, ***p <.001

MDA, T-SOD activity, and T-GSH levels in the supernatants obtained from the PFC were analyzed. The difference in MDA levels among the groups was statistically significant (Kruskal-Wallis, *H*(7) = 42.439, *p* < 0.001). MDA levels were greater in the F-VPA group than in the F-Control group (*p* = 0.004) and in the M-VPA group than in the M-Control group (*p* < 0.001). MDA levels were lower in the F-VPA+MOTS-c group than in the F-VPA group (*p* = 0.005) and in the M-VPA+MOTS-c group than in the M-VPA group (*p* = 0.048) (Fig. 11A). The difference in T-SOD activity among the groups was also statistically significant (Kruskal-Wallis, *H*(7) = 31.042, *p* < 0.001). T-SOD activity was lower in the F-VPA group than in the F-Control group (*p* = 0.040) and in the M-VPA group than in the M-Control group (*p* = 0.009). T-SOD activity was higher in the F-VPA+MOTS-c group than in the F-VPA group (*p* < 0.001) and in the M-VPA+MOTS-c group than in the M-VPA group (*p* < 0.001) (Fig. 11B). The difference in T-GSH levels among the groups was statistically significant (one-way ANOVA, *F*(7, 56) = 14.082, *p* < 0.001). T-GSH levels were lower in the F-VPA group than in the F-Control group (*p* < 0.001) and in the M-VPA group than in the M-Control group (*p* < 0.001). T-GSH levels were higher in the F-VPA+MOTS-c group than in the F-VPA group (*p* = 0.010) and in the M-VPA+MOTS-c group than in the M-VPA group (*p* = 0.024) (Fig. 11C).

### BH4 and BDNF

No statistically significant differences were found among the groups in terms of plasma BH4 (Kruskal-Wallis, *H*(7) = 3.830, *p* = 0.799) or BDNF (one-way ANOVA, *F*(7, 56) = 1.634, *p* = 0.145) levels (Fig. 11E and F).

## Discussion

When studies utilizing the VPA-induced autism model are reviewed, conflicting results are noteworthy. Some studies have reported changes in opposite directions, whereas others have reported no observable alterations in parameters such as social behavior, repetitive behavior, locomotor activity, and anxiety following VPA exposure. Similarly, contradictory findings have been reported regarding sex-related effects. Therefore, differences in factors such as study design, timing, and dosage of VPA administration, and the species and age of the animals tested are crucial considerations when comparing results [56].

### MOTS-c Improves Core Symptoms of Autism

Reduced social interaction is one of the two core symptoms of ASD [1]. The three-chamber sociability test is a frequently used method designed to assess social interaction in experimental animals [57, 58]. In the sociability phase of the test, the preference of the test animal for a novel rat over an object (SI) is evaluated, while in the social novelty phase, the preference for a novel rat over a familiar one (SNI) is assessed [57, 59]. In studies evaluating changes in social behavior in the VPA-induced autism model, some have reported a decrease in sociability in both sexes [60, 61], whereas others have shown reduced sociability predominantly in male animals, with no significant reduction observed in females, suggesting that social impairment may be less pronounced in the female sex [62, 63]. In our study, significant differences were observed among the groups in both the SI and the SNI, which were used as indicators of social behavior. Our results demonstrate that social interaction was reduced in the VPA groups of both sexes compared with the Control groups. MOTS-c treatment in VPA-exposed rats reversed this reduction by enhancing social interaction parameters.

The other core symptom of ASD is stereotyped repetitive behaviors [1]. Various tests are used in animal studies to assess repetitive behaviors, among which the Y maze and marble burying tests were employed in this study. In addition to its use in evaluating different memory functions, the Y maze is frequently used to assess repetitive behaviors in animal models of autism [47, 64]. In studies where only male offspring were assessed, the SA, considered an indicator of repetitive behavior, was found to be lower in the VPA groups than in the control groups [40, 47, 64]. Another test commonly used to evaluate repetitive behavior in autism models is the marble burying test. It has been suggested that increases in repetitive behaviors are reflected by elevated burying behavior and a greater number of buried marbles in this test. Previous studies have shown that prenatal exposure to VPA leads to an increased number of buried marbles in male offspring [10, 65]. According to our results, the lower SA in the Y maze and the greater number of buried marbles in the marble burying test observed in VPA-exposed rats are indicative of increased repetitive behavior. MOTS-c treatment attenuated these VPA-induced repetitive behavior symptoms in both sexes.

### Effects of VPA and MOTS-c on Locomotor Activity and Anxiety

Human and animal studies have demonstrated increased locomotor activity [63] and anxiety in ASD [66, 67]. The open field test is a widely used method in animal studies to evaluate both locomotor activity and anxiety levels [44, 68]. Several studies have reported that the distance traveled [61] and immobility time, as evaluated in the open field test, did not differ between the VPA and control groups in either sex [56]. Similarly, in our study, no significant effects of VPA or MOTS-c were observed on the distance traveled or the immobility time in the open field test, which are used as indicators of locomotor activity. Studies assessing locomotor activity using the open field test and other methods have reported higher locomotor activity levels in female rats than in males, based on group-level comparisons [44, 69, 70]. Our findings are also compatible with this observation.

In the open field test, reduced time spent in the center area and increased time spent in the periphery are interpreted as indicators of elevated anxiety. There are conflicting results regarding the effects of VPA on anxiety-related behaviors. For example, one study reported no effect of VPA on the time spent in the center area [56], whereas another reported that prenatal VPA exposure increased anxiety in both sexes [61]. However, another study reported increased anxiety only in males following VPA exposure [69]. Our findings indicate that VPA exposure was associated with significant anxiety-like behavioral changes in male rats, whereas corresponding differences did not reach statistical significance in females. Moreover, MOTS-c treatment did not significantly affect the time spent in the center in either sex.

In our study, the time spent in an active state was determined via pixel-based analysis of the video tracking software. To our knowledge, no previous study has assessed active time via a similar method in the VPA-induced autism model. Although no significant changes were detected in standard locomotor activity parameters, the increase in active time, particularly in male VPA-exposed rats, may be associated with elevated anxiety levels. Although MOTS-c treatment reduced the time spent in the active state, it did not significantly alter the time spent in the center area. These findings suggest that the dose of MOTS-c administered in this study might have been insufficient to fully alleviate anxiety-related symptoms.

### Cerebellum Plays a Significant Role in the Pathophysiology of ASD

Various human and animal studies have reported that ASD leads to alterations in multiple brain regions [8]. Most of these studies have focused on the cerebellum, hippocampus, and PFC. In these regions, neuronal disorganization, neuroinflammation, neurodegeneration, and synaptopathies have been identified [55, 71]. Cerebellar pathologies have been associated with impairments in social behavior and motor functions, as well as increased anxiety; hippocampal pathologies, with deficits in memory and learning; and PFC abnormalities, with challenges in social behavior and communication [47, 55, 72].

The function of the cerebellum is traditionally known as the control and coordination of movement. In addition, numerous studies have demonstrated that it also plays a role in movement planning, motor learning, timing, language processing, and emotional, cognitive, and social functions. Histologically, the cerebellar cortex consists of three layers from the surface to the white matter: (I) the molecular layer, (II) the Purkinje cell layer, and (III) the granular layer. Purkinje cells are large neurons with extensive dendritic arborization that are arranged in a single row between the molecular and granular layers. They are the only efferent neurons of the cerebellar cortex and are predominantly inhibitory in function [73].

Clinical evidence, neuroimaging studies, animal models, and postmortem findings collectively suggest that cerebellar dysfunction plays a significant role in the pathophysiology of ASD. In many animal models of autism, abnormalities in the cerebellar cortex have been observed [74]. Imaging and postmortem studies in individuals with autism have consistently revealed three major cerebellum-related abnormalities: (I) a reduction in the number of Purkinje cells, (II) cerebellar atrophy, and (III) disruptions in the neural circuits connecting the cerebellum with other brain regions, such as the thalamus, pons, and cortex [73]. Similarly, in VPA-induced animal models of autism, a reduction in the number of Purkinje cells, although with some region-specific variations, has been reported in both sexes. Additionally, cerebellar volume loss and abnormalities in the dendritic arborization of Purkinje cells have been detected in these models [15, 73, 75, 76]. Considering the known effects of cerebellar pathologies, the reduction in Purkinje cell number detected in both male and female VPA groups in our study may be associated with the alterations observed in social interaction and anxiety-related behaviors. Taken together with previous findings from both human and animal studies, our results further underscore the significant role of the cerebellum in ASD pathophysiology.

The main cause and underlying mechanisms of Purkinje cell damage in the cerebellum remain incompletely understood. It has been hypothesized that, owing to their large size and high metabolic activity, Purkinje cells are more vulnerable to damage [77]. In addition to the reduction in Purkinje cell numbers, studies in animals prenatally exposed to VPA have reported structural alterations, such as shrinkage of the cell body, eosinophilic cytoplasm, enlarged perineuronal spaces, and shrunken or barely distinguishable nuclei [10, 78, 79]. These changes have been attributed to increased oxidative stress induced by VPA exposure [78]. Consistent with these findings, our study also demonstrated that, along with a reduction in Purkinje cell numbers, rats administered VPA presented histopathological changes in Purkinje cells, including shrinkage of the cell body, prominent degeneration, and markedly enlarged perineuronal spaces.

### MOTS-c Treatment Prevents Purkinje Cell Loss

In this study, MOTS-c treatment was shown to reduce Purkinje cell damage and prevent Purkinje cell loss in VPA-administered rats. Increased oxidative stress was observed in the PFC of both male and female rats prenatally exposed to VPA, and MOTS-c exerted antioxidant effects in this region. Although oxidative stress markers in the cerebellum could not be evaluated, the protective effect of MOTS-c, known for its antioxidant properties, on Purkinje cells suggests that the observed damage may be related to increased oxidative stress.

Purkinje cells appear to play a significant role in the pathophysiology of ASD. Several hypotheses have been proposed that the number of Purkinje cells in ASD may be reduced through different mechanisms during their generation, migration, or maturation phases [80]. For example, a postmortem human study suggested that in individuals with autism, Purkinje cells may be lost after their generation and migration are complete [81]. In rats, Purkinje cells begin migrating from the primitive cerebellar neuroepithelium to the cortex around ED14, and this migration is completed approximately 2–3 days before birth. From that point onward, the Purkinje cell maturation process begins and continues until PND30, a process influenced by both intrinsic and extrinsic signals [82]. In our study, MOTS-c treatment was administered between PND21 and PND46 following prenatal VPA exposure at ED12. Histological evaluations revealed that the Purkinje cell numbers in the MOTS-c-treated groups approached normal levels. Therefore, MOTS-c may have exerted protective effects on VPA-induced Purkinje cell damage, particularly during and after the maturation phase.

The finding that MOTS-c treatment increased Purkinje cell numbers to near-normal levels in VPA-exposed rats is particularly noteworthy. Oxidative stress caused by increased reactive oxygen species (ROS) can lead to cell death [83, 84]. However, it remains unclear at which phase the oxidative stress induced by VPA results in Purkinje cell damage and loss. If this loss occurs predominantly after PND21, the antioxidant properties of MOTS-c may have helped to prevent this damage. Conversely, if most Purkinje cell loss occurs before PND21, this protective effect may not be explained by the antioxidative action of MOTS-c. At this point, it is possible to speculate that MOTS-c might induce neurogenesis in Purkinje cells. Neurogenesis in specific regions of the adult brain, such as the olfactory bulb and the dentate gyrus, has been reported, and research in this area is ongoing. Neurogenesis in the cerebellum has also been proposed, but its origin remains poorly understood [85]. Under physiological conditions, Nestin-expressing progenitor cells in the cerebellum are suggested to give rise to interneurons and astroglial cells, and in response to cerebellar injury, they may differentiate into mature granule neurons [85–87]. Although these studies provide evidence for granule cell neurogenesis in the cerebellum, there is currently no definitive evidence supporting the neurogenesis of Purkinje cells.

### Neuropathological Findings in the PFC

The PFC is a cerebral cortex area responsible for the regulation of cognitive functions and is thought to play an important role in the pathophysiology of ASD [8]. Although the PFC has a complex structure in the human brain, it is not as fully developed in rodents. Therefore, this distinction should be taken into account when the findings are interpreted. The cingulate cortex, infralimbic cortex, and prelimbic cortex, located in the anterior part of the rat brain, are considered components of the PFC (Fig. 3) [8]. Previous studies have reported neuropathological alterations in the PFC of individuals with ASD and animal models, including neuronal disorganization and dysregulation, changes in pyramidal neuron morphology, and excitatory/inhibitory imbalance [8, 88]. In a study investigating the effects of prenatal VPA exposure, evidence of neurodegeneration and apoptosis was observed in the PFC, hippocampus, and cerebellum [10]. In a mouse study that included both sexes, postnatal VPA exposure led to widespread cortical damage in male offspring, whereas in females, the damage was localized primarily to the PFC. The same study noted that histopathological findings in the PFC and Purkinje cells were similar in both sexes [76]. In our study, similar histopathological alterations were observed in the PFC and cerebellum across male and female groups.

In a study involving only male offspring, increased neuronal damage scores, darkly stained and pyknotic nuclei containing degenerated neurons, neuronal disorganization, and apoptosis were reported in the VPA group [72]. Similarly, another study evaluating the PFC revealed increased histopathological scores, along with neuronal damage, neuronal loss, and pyknosis, in the VPA group. In that study, which investigated the effects of homotaurine, a compound with anti-inflammatory and antioxidant properties, high doses of homotaurine were shown to reverse VPA-induced damage to the PFC. Homotaurine was also reported to exert neuroprotective effects on the cerebellum [15]. In our study, MOTS-c treatment led to a decreasing trend in neuronal damage in the PFC of VPA-exposed rats; however, this effect was not statistically significant. This finding may be related to the dose used, and higher doses of MOTS-c might yield statistically significant effects. In previous animal studies, MOTS-c was typically administered at a dose of 5 mg/kg (i.p.) for short durations (4–7 days) or 0.5 mg/kg (i.p.) for longer durations (3–8 weeks) [18]. Therefore, 0.5 mg/kg MOTS-c was administered i.p. between PND21 and PND46 (for 26 days) in this study.

### MOTS-c Exerts Antioxidant Effects in the PFC

Oxidative stress can be defined as cellular damage induced by free radicals such as ROS and reactive nitrogen species (RNS). Under physiological conditions, a dynamic balance exists between intracellular ROS/RNS production and the antioxidant defense system. Oxidative stress occurs when the level of ROS/RNS exceeds the antioxidant capacity. Elevated ROS levels interact with lipids, proteins, and nucleic acids within the cell, and disruption of the balance between oxidants and antioxidants ultimately leads to cell death [89–92].

Lipid peroxides and hydrocarbon polymers with toxic properties are generated when polyunsaturated fatty acids (PUFAs) react with ROS. MDA, a terminal product of PUFA peroxidation, is commonly used as a biomarker for lipid peroxidation. Increased lipid peroxidation has been reported in the plasma of children with autism compared with that in their healthy siblings. Other studies have also shown elevated levels of other lipid peroxidation markers in autism [90]. Additionally, studies have demonstrated that biomarkers of lipid peroxidation, protein oxidation, and DNA oxidation are elevated in both the blood and brain tissues of individuals with autism. Concurrently, reduced plasma levels of reduced glutathione (GSH) and T-GSH, along with increased GSSG levels, have been reported. GSH functions as a physiological antioxidant that protects cells against free radicals. Owing to lower plasma GSH levels, these children are considered to be more susceptible to oxidative stress [91].

Many rat studies have reported that male offspring exposed to VPA exhibit elevated oxidative stress markers (e.g., MDA and TBA-reactive substances), decreased activities of antioxidant enzymes (e.g., glutathione peroxidase, superoxide dismutase [SOD], and catalase [CAT]), reduced GSH levels, and increased levels of proinflammatory cytokines (e.g., IL-1β, IL-6, and TNF-α) in various central nervous system regions, such as the PFC, cerebellum, and hippocampus [15, 47, 54, 55, 72, 93]. In a rat study involving both sexes, compared with control groups, the VPA groups presented lower total antioxidant capacity, CAT activity, SOD activity, and anti-inflammatory cytokine IL-10 levels, whereas the levels of MDA, protein carbonyl (a marker of protein oxidation), and the proinflammatory cytokines IL-1β and IL-6 were elevated in brain tissue. Moreover, the levels of oxidative and inflammatory markers were found to be greater in male VPA groups than in female VPA groups [94]. Our results further support that VPA induces oxidative stress in the PFC in both sexes. Notably, MOTS-c treatment reversed these alterations by enhancing antioxidant capacity and attenuating oxidative damage, consistent with its reported antioxidative properties.

### Beneficial Effects of MOTS-c Are Independent of BH4 and BDNF

BH4 acts as a cofactor for several enzymes involved in critical metabolic pathways, including monoamine neurotransmitter synthesis, phenylalanine degradation, and nitric oxide (NO) production [95]. One of these enzymes is nitric oxide synthase (NOS). In the presence of sufficient BH4, NOS monomers dimerize to produce NO. However, when BH4 levels are inadequate, NOS monomers fail to couple, and free radicals are formed instead of NO [96, 97].

In children diagnosed with autism, cerebrospinal fluid (CSF) BH4 levels were found to be 42% lower than those in controls [36]. Another study reported no significant differences in plasma and urinary BH4 levels between autism and control groups [98]. In a clinical trial, BH4, which is known to cross the BBB [38], was administered to children aged 3–7 years with autism, and a regression of autism symptoms was reported in the treatment group [99, 100].

BDNF, which is highly expressed in the brain, is a neurotrophin that plays a key role in neuronal differentiation and synaptic plasticity. It has been suggested that abnormalities in BDNF-related signaling mechanisms play a significant role in the pathophysiology of many psychiatric and neurological disorders. Since BDNF can cross the BBB bidirectionally, blood levels of BDNF are considered to reflect its brain concentrations [37, 101, 102]. Studies on individuals with autism have reported conflicting findings regarding blood BDNF levels, with some showing increased levels, others showing decreased levels, and some reporting no difference compared with healthy controls. Similarly, findings from animal studies are also variable [102, 103]. For example, increased BDNF expression was observed in fetal brains following prenatal VPA exposure in mice [104], whereas other rodent studies have reported decreased BDNF levels in various brain regions, such as the cingulate cortex, cerebellum, hippocampus, and PFC, in male offspring exposed to VPA in utero [37, 102, 105–107]. Given the assumption that brain BDNF levels are reflected in blood circulation, our findings suggest that VPA exposure does not significantly affect brain BDNF levels. Nonetheless, central changes in BDNF may not have been detected in the peripheral circulation.

One previous study reported that MOTS-c is unable to cross the BBB [30]. Accordingly, in our study, MOTS-c was thought to exert its effects indirectly through peripheral molecules capable of crossing the BBB. MOTS-c has been shown to increase AMPK activity [19], and AMPK activation has been reported to increase BH4 and BDNF levels, both of which have antioxidative effects [31, 32]. Moreover, exercise reportedly improves autistic symptoms, enhances AMPK activity, increases BH4 and BDNF levels, and reduces oxidative stress in the brain [26, 33–35]. Additionally, reduced BH4 and BDNF levels have been reported in individuals with autism [36, 37]. On the basis of these findings, in this study, it was hypothesized that the MOTS-c peptide, which mimics the effects of exercise, might increase circulating BH4 and BDNF levels through AMPK activation. These molecules could then reduce oxidative stress in the central nervous system and improve autism-like symptoms. However, our findings showed that MOTS-c administration did not alter plasma BH4 or BDNF levels. Nevertheless, i.p. administration of MOTS-c ameliorated autism-like behaviors in VPA-exposed rats, induced antioxidant effects in the PFC, appeared to have a neuroprotective effect against neuronal damage, and restored Purkinje cell numbers in the cerebellum to near-normal levels. These results suggest that the therapeutic effects of MOTS-c in the autism model may occur independently of circulating BH4 and BDNF.

Notably, our plasma BH4 and BDNF measurements reflect only the chronic effects of MOTS-c. Acute exercise has been shown to transiently increase circulating BDNF levels [108]. Therefore, MOTS-c might also have induced transient changes in BH4 or BDNF levels. Furthermore, given the central role of AMPK in regulating various metabolic pathways [109], MOTS-c might exert its beneficial effects via other circulating molecules capable of crossing the BBB. Additionally, the increased BBB permeability induced by VPA [110] might have facilitated the passage of MOTS-c into the central nervous system.

## Conclusion

Prenatal VPA exposure led to impaired sociability, repetitive behaviors, anxiety-like responses, cerebellar Purkinje cell loss, and increased oxidative stress and neuronal damage in the PFC. MOTS-c treatment ameliorated histopathological, biochemical, and behavioral alterations, except for anxiety-related behaviors and neocortical damage. No significant changes were detected in plasma BH4 or BDNF levels. Collectively, these findings indicate that MOTS-c exerts neuroprotective and antioxidant effects in the VPA-induced autism model through mechanisms independent of circulating BH4 and BDNF, suggesting its potential as a therapeutic candidate for ASD.

## Limitations and Future Perspectives

This study has several limitations. The most notable limitation is that only a single dose of MOTS-c was tested. The selected dose might have been insufficient to fully reveal the peptide’s potential behavioral and molecular effects. Therefore, dose-response experiments are needed to determine whether higher doses of MOTS-c could produce more pronounced or differential outcomes. Future studies investigating the effects of MOTS-c at different doses and durations in autism animal models are warranted to clarify this issue and to determine whether the non-significant changes observed in some parameters were dose-related.

Although both male and female animals were included and analyzed as separate experimental groups, the statistical approach was not designed to formally test sex-specific effects or sex × treatment interactions. Single-factor group–based analysis allowed the identification of sex-related differences across all experimental groups; however, this approach does not provide a comprehensive assessment of interaction effects that would require a factorial statistical framework. Future studies specifically designed to evaluate sex × treatment interactions will be necessary to more rigorously characterize sex-dependent responses to MOTS-c.

Another limitation of this study is that oxidative stress parameters in the cerebellum could not be evaluated. This limitation prevented us from directly linking oxidative imbalance to Purkinje cell damage. Although our study demonstrated a significant loss of Purkinje cells in the cerebellum and the protective effects of MOTS-c treatment, we were unable to determine the specific developmental period during which this neuronal loss occurred. Consequently, it remains unclear through which mechanisms MOTS-c reverses or prevents Purkinje cell degeneration. In this context, further research is needed to determine the critical period during which VPA-induced Purkinje cell damage is most prominent and to elucidate the mechanisms by which MOTS-c exerts its neuroprotective effects on Purkinje cell numbers.

Although several human studies have examined BH4 in the context of autism, to our knowledge, no previous animal studies have assessed BH4 levels in blood or brain tissue in autism models. Despite its important role in oxidative/nitrosative stress and monoamine neurotransmitter synthesis, the contribution of BH4 to the pathophysiology of ASD remains incompletely understood. Further research, particularly those focusing on BH4 levels in the brain, is needed to clarify its potential role in ASD.

The potential mechanisms through which MOTS-c may exert its effects, as discussed in the “Discussion” section, were not directly evaluated within the scope of this study. In particular, acute and time-dependent changes in BH4 and BDNF levels, alternative AMPK-related molecular pathways, and the contribution of VPA-induced alterations in BBB integrity to treatment responses were not assessed. Further studies are needed to elucidate these mechanisms in detail.

## Data Availability

The datasets generated and/or analyzed during the current study are available in the TÜBİTAK Aperta repository, 10.48623/aperta.274267. Access to the data is restricted but can be granted upon a reasonable request to the corresponding author.

## Declarations

This study was conducted as part of the medical specialty thesis of the corresponding author and was completed at the Department of Physiology, Cerrahpaşa Medical Faculty, İstanbul University-Cerrahpaşa.
